# Heterozygous Mutations of *FREM1* Are Associated with an Increased Risk of Isolated Metopic Craniosynostosis in Humans and Mice

**DOI:** 10.1371/journal.pgen.1002278

**Published:** 2011-09-08

**Authors:** Lisenka E. L. M. Vissers, Timothy C. Cox, A. Murat Maga, Kieran M. Short, Fenny Wiradjaja, Irene M. Janssen, Fernanda Jehee, Debora Bertola, Jia Liu, Garima Yagnik, Kiyotoshi Sekiguchi, Daiji Kiyozumi, Hans van Bokhoven, Carlo Marcelis, Michael L. Cunningham, Peter J. Anderson, Simeon A. Boyadjiev, Maria Rita Passos-Bueno, Joris A. Veltman, Ian Smyth, Michael F. Buckley, Tony Roscioli

**Affiliations:** 1 Department of Human Genetics, Nijmegen Centre for Molecular Life Sciences, Radboud University Nijmegen Medical Centre, Nijmegen, The Netherlands; 2 Division of Craniofacial Medicine, Department of Pediatrics, University of Washington, Seattle, Washington, United States of America; 3 Department of Anatomy and Developmental Biology, Monash University, Clayton, Australia; 4 Department of Biochemistry and Molecular Biology, Monash University, Clayton, Australia; 5 Centro de Estudos do Genoma Humano, Instituto de Biociências, Universidade de São Paulo, São Paulo, Brasil; 6 Section of Genetics, Department of Pediatrics, University of California Davis, Sacramento, California, United States of America; 7 Institute for Protein Research, Osaka University, Osaka, Japan; 8 Donders Institute for Brain, Imaging, and Cognition, Nijmegen, The Netherlands; 9 Australian Craniofacial Unit, Women and Children's Hospital, Adelaide, Australia; 10 Department of Haematology and Genetics, South-Eastern Area Laboratory Services, Prince of Wales and Sydney Children's Hospitals, Randwick, Australia; 11 Sydney South West Genetic Service, Royal Prince Alfred Hospital, Sydney University, Sydney, Australia; Medical Research Council Human Genetics Unit, United Kingdom

## Abstract

The premature fusion of the paired frontal bones results in metopic craniosynostosis (MC) and gives rise to the clinical phenotype of trigonocephaly. Deletions of chromosome 9p22.3 are well described as a cause of MC with variably penetrant midface hypoplasia. In order to identify the gene responsible for the trigonocephaly component of the 9p22.3 syndrome, a cohort of 109 patients were assessed by high-resolution arrays and MLPA for copy number variations (CNVs) involving 9p22. Five CNVs involving *FREM1*, all of which were *de novo* variants, were identified by array-based analyses. The remaining 104 patients with MC were then subjected to targeted *FREM1* gene re-sequencing, which identified 3 further mutant alleles, one of which was *de novo*. Consistent with a pathogenic role, mouse *Frem1* mRNA and protein expression was demonstrated in the metopic suture as well as in the pericranium and dura mater. Micro-computed tomography based analyses of the mouse posterior frontal (PF) suture, the human metopic suture equivalent, revealed advanced fusion in all mice homozygous for either of two different *Frem1* mutant alleles, while heterozygotes exhibited variably penetrant PF suture anomalies. Gene dosage-related penetrance of midfacial hypoplasia was also evident in the *Frem1* mutants. These data suggest that CNVs and mutations involving *FREM1* can be identified in a significant percentage of people with MC with or without midface hypoplasia. Furthermore, we present *Frem1* mutant mice as the first *bona fide* mouse model of human metopic craniosynostosis and a new model for midfacial hypoplasia.

## Author Summary

Although twin and family studies have shown that genes play a critical role in the timing of fusion of skull bones, the identification of specific genes that may be involved has remained somewhat elusive except in the case of the dominantly inherited craniosynostosis syndromes. Metopic craniosynostosis (MC), the early fusion of the forehead (frontal) bones, accounts for 5%–15% of all craniosynostosis cases. This premature fusion of the frontal bones results in a characteristically altered skull shape, termed trigonocephaly, that usually requires surgical correction. Remarkably, the cause of the majority of cases of MC remains unknown (idiopathic). Here, we report genetic variants involving chromosome 9 which involve and interrupt the structure of the *FREM1* gene in a large cohort of patients presenting with unisutural metopic craniosynostosis. Micro-computed tomographic (microCT) imaging and quantitative analysis of skull shape reveal both premature fusion of the PF suture (metopic equivalent) and also changes in frontal bone shape supportive of a role for Frem1 in regulation of the metopic suture. Taken together with Frem1 gene and protein expression findings, these data indicate that mutations in *FREM1* can give rise to metopic craniosynostosis.

## Introduction

During development the calvarial bones are separated by regions of non-calcified intrasutural mesenchyme which permit skull deformation during birth and accommodate brain growth during childhood. Craniofacial malformations caused by the premature fusion of cranial sutures are common presenting features in clinical genetics practice with an overall incidence of approximately 1 in 2500 live births. Non-syndromic forms of craniosynostosis predominate, but there are more than 90 described syndromic craniosynostoses which are conventionally classified by their pattern of suture involvement and dysmorphic features [1]. Syndromic craniosynostoses have been shown to arise from at least 6 different mechanisms; activation of receptor kinase signaling pathways (*FGFR1-3*, *TGFBR1-2*, *EFNB1*), inactivation of transcription factors (*TWIST1*, *MSX2*), alterations to, or interactions with, extracellular matrix proteins (*FBN1*, *FGF9*), and mutations of ER proteins (*POR*), helicases (*RECQL4*) or membrane trafficking proteins (*RAB23*, *SEC23A*) [2]. In contrast, the genetic etiologies of the non-syndromic craniosynostoses are understood poorly. Metopic craniosynostosis (MC) resulting in trigonocephaly occurs predominantly as a non-syndromic craniosynostosis with an estimated prevalence of between 1∶15-68,000 live births [3], [4]. A number of reports suggest that MC has been under-diagnosed as the frequency of identification is rising in multidisciplinary craniofacial units [3], [5]. Review of the craniosynostosis literature, OMIM and the London Dysmorphology database identifies more than 70 conditions that can exhibit trigonocephaly/MC as a clinical feature (Table S1). Syndromes with MC may be sub-classified into seven main etiological groups including: common craniofacial syndromes related to known craniofacial genes (*FGFR* family, *TWIST*); other recognized clinical genetic syndromes resulting in microcephaly/microencephaly; developmental disorders of the midline; recurrent chromosomal/genomic copy number imbalances; syndromes with abnormal metabolic function; teratogenic/environmental causes; and an idiopathic group (Table S1).

One of the better characterized forms of MC is associated with monosomy for an 8Mb interval of chromosome 9p22.3 (OMIM 158170). The major clinical features of this contiguous gene deletion syndrome are mental retardation, trigonocephaly, midface hypoplasia and a long philtrum [6]–[10]. Swinkels et al., mapped the trigonocephaly component by array-CGH to a genomic interval of 300 kb [11]. This region partially overlaps the interstitial deletion identified by Kawara et al., in a patient with trigonocephaly who had a complex cytogenetic re-arrangement [12]. Jehee et al., sequenced *CER1,* a candidate gene located within that interval, but failed to identify any pathogenic changes in a cohort of 70 syndromic and non-syndromic trigonocephaly patients [13]. It is noteworthy that *CER1* is located immediately telomeric to the interval described by Swinkels et al., and that another interesting candidate gene, *FREM1,* is in close proximity to the breakpoints defined previously [11].

The role of intrasutural mesenchyme extracellular matrix (ECM) components in determining normal sutural development is underscored by the frequent cranial suture involvement in Marfan syndrome and the demonstration that proteoglycan/FGF9 interactions regulate sutural growth factor concentrations [14]. Because of this, we became interested in *FREM1,* which resides in the 300 kb interval defined by Swinkels et al [11] as a candidate for metopic craniosynostosis. FREM1 is a secreted protein of mesenchymal cells which forms a ternary complex with the epithelial cell integral membrane proteins, FRAS1 and FREM2, mutations in which have been found in patients with Fraser syndrome (OMIM 219000) whose clinical phenotype includes craniofacial dysmorphism. FREM1 is expressed in regions of epidermal/mesenchymal interaction and remodelling and shows notable embryonic expression in midline structures [15]. Homozygous recessive mutations in *FREM1* have been identified recently in two rare conditions: BNAR syndrome which is characterized by bifid nose, anorectal malformations and renal agenesis (OMIM 608980) [15] and; Manitoba-Oculo-Tricho-Anal (MOTA) syndrome (OMIM 248450), which is characterized by a bifid or broad nasal tip, eye colobomas, cryptophthalmos and anophthalmia/microphthalmia, aberrant hairline and anal stenosis [16].

In this report we provide evidence that mutations in *FREM1* can also be associated with trigonocephaly. We have identified 8 mutant alleles of human *FREM1* in a variety of mutational classes including structural variants that interrupt the *FREM1* gene, CNVs of the entire *FREM1* locus and point mutations of the *FREM1* coding sequence. These results are supported by *Frem1* gene expression studies, suture imaging and quantitative analysis of craniofacial shape in heterozygous and homozygous *Frem1* mutant mice.

## Results

The initial stage of our analysis was to re-examine the CNV described in the three patients defining the 300 kb trigonocephaly interval reported by Swinkels et al., using high density chromosome 9 specific arrays (patients 1-3; [11]). These results re-defined the CNV boundaries in these patients compared with the results obtained with lower resolution BAC arrays. Furthermore, an additional patient with MC had been ascertained subsequent to the report of Swinkels et al., who was found to have an interstitial CNV of distal 9p (patient 4). Genomic analyses in these four subjects re-defined the region contributing to MC in the 9p deletion syndrome.

### CNVs in patients with MC disrupt the *FREM1* gene

**Figure 1:**
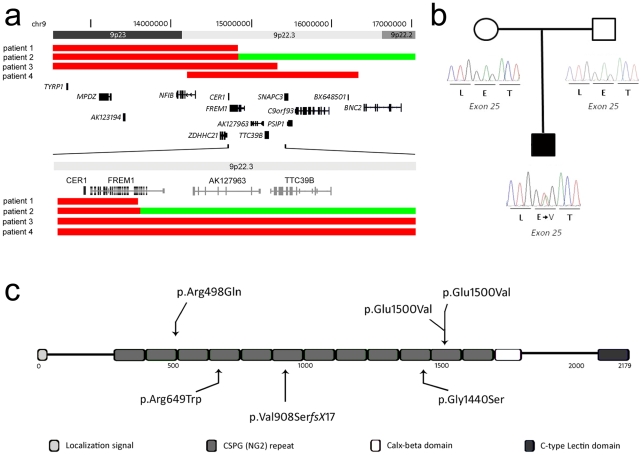
Schematic overview of CNVs and mutations affecting the *FREM1* gene. (a) Deletions in patients 1-4 are represented by solid red bars, whereas the duplicated segment in patient 2 is represented by a solid green bar. The proximal deletion breakpoint of patient 1 as well as the deletion/duplication breakpoint in patient 2 disrupts *FREM1.* (b) Partial electropherograms of the *de novo* mutated nucleotide identified in patient 6. (c) Schematic overview of the FREM1 protein showing the domain structure and positions of mutations in the FREM1-trigonocephaly (above) and BNAR (below) syndromes.

The CNVs identified in each of these four patients are summarised in Figure 1a, Table 1 and are as follows. Re-analysis of two previously reported CNVs identified that they contained deletion breakpoints within the *FREM1* gene. Patient 1 was shown to have a 14.83 Mb deletion which extended from *FREM1* intron 9 to the chromosome 9p telomere, deleting exons 10-37 of *FREM1*. Patient 2 had a complex chromosome 9 deletion-duplication mutation with a breakpoint within *FREM1*. The deletion component of this re-arrangement extended from *FREM1* intron 6 to the 9p telomere, deleting exons 7-37 of *FREM1.* The *FREM1* sequence immediately centromeric to the deletion formed a 37 Mb duplication extending between genomic co-ordinates 14.84 Mb and 51.8 Mb [hg18].

**Table 1:** Molecular and clinical patient information.

	*Patients with CNVs affecting FREM1*					*Patients with mutations affecting FREM1*		
	Patient 1*	Patient 2*	Patient 3*	Patient 4*	Patient 5	Patient 6	Patient 7	Patient 8
***FREM1 mutation***	Del ex10-37	Dup ex1-6; Del ex7-37	Del	Del	Del	c.4499A>T; p.Glu1500Val	c.4499A>T; p.Glu1500Val	c.1493G>A; p.Arg498Gln
***Inheritance***	*de novo*	*de novo*	*de novo*	*de novo*	*de novo*	*de novo*	maternally inherited	paternally inherited
***General Clinical features***								
*developmental delay*	+	+	+	+	+	+	−	−
*speech delay*	+	+	+	+	+	NA	−	−
*motor delay*	+	unknown	+	−	−	NA	−	±
*hypotonia*	+	+	−	−	−	NA	−	±
*height*	-1 SD	-2 SD	-1.5SD	-1.5 SD	-1SD	NA	-1SD	−
***Head***								
*trigonocephaly*	+	+	+	+	+	+	+	+
*midface hypoplasia*	+	±	+	+	+	NA	±	−
*eyes upward slant*	−	+	+	−	+	NA		−
*downward slant*	−	−	−	−	−	NA	+	−
*short palpebral fissure*	+	+	±	−	unknown	NA	−	−
*epicanthic folds*	−	+	−	+	−	NA	±	−
*high arched eyebrows*	−	−	+	−	+	NA	−	−
*head circumference*	unknown	unknown	0 SD	0 SD	unknown	NA	-3SD	−
*nose*	short/flat	short/flat	short/flat	short	short	NA	Broad bridge	Flat nasal bridge
***Additional clinical features***								
*renal abnormalities*	−	−	−	−	−	NA	Mild Right Pelvicaliceal dilatation	−
*abnormal genitalia*	−	−	cryptorchidism	−	−	NA	−	−
								
*inguinal hernia*	−	−	+	+	−	NA	−	−
*cardiac malformations*	−	−	PS/I	PPS	VSD	+	−	−
*other*	−	−	−	−	−	−	Hypoglycemic neonatal seizures	−

*More detailed clinical descriptions can be found in Swinkels et al. (ref11); del, deletion; dup, duplication; ex, exon; SD, standard deviation; PS/I. Pulmonary stenosis/incompetence; PPS, peripheral pulmonary stenosis; VSD. Ventriculo-septal defect ; +, present; −, absent ; NA, not available.

The third CNV was a 15.32 Mb deletion in Patient 3 which extended from the chromosome 9p telomere to the intergenic region between *TTC39B* and *SNAPC3* resulting in the deletion of the entire *FREM1* locus. Patient 4 had the smallest CNV; a 2.14 Mb interstitial 9p deletion between genomic co-ordinates 14.20 and 16.34 Mb [hg18] which involved only 9 genes. This region extends from *NFIB* intron 2 to the intergenic region between *C9orf93* and *BNC2* also deleting *FREM1*. All CNVs were confirmed by MLPA and were shown to be *de novo*.

The common 0.65 Mb region of deletion overlap identified in the four samples contains 4 genes *NFIB*, *ZDHHC21*, *CER1* and *FREM1*. Importantly two patients were identified to have CNVs whose boundaries disrupt the coding region of the *FREM1* gene. These CNV data are consistent with a model in which haploinsufficiency of *FREM1* is associated with MC. These findings prompted us to investigate whether DNA sequence analysis of *FREM1* might identify additional pathogenic mutations.

Prior to re-sequencing of the 35 coding exons of *FREM1* (see below), the 105 additional patients with MC were pre-screened for point mutations in *CER1* and deletions of *FREM1*. One further patient (patient 5) was confirmed to have a whole *FREM1* gene deletion as a result of a cytogenetically visible 9p22.3 deletion. No mutations were identified in *CER1,* consistent with previous reports [13].

### Missense mutations in *FREM1* in patients with MC

104 patients with a clinical diagnosis of MC were screened for DNA sequence mutations in *FREM1*. Two previously unreported nucleotide substitutions were identified in three patients (Table 1; Figure 1c). The first of these mutations, NM_144966.4:c.4499A>T [NP_659403.4: p.(Glu1500Val)] in exon 25, was observed in two unrelated patients (patient 6 and patient 7). p.Glu1500Val was also present in patient 6 as a *de novo* event (Figure 1b) consistent with both his parents being clinically normal. The Grantham score for this substitution is substantial at 121 and the mutation is predicted not to be tolerated by SIFT, and possibly damaging by Polyphen2 [HumVar] with a score of 0.309 [17]–[19]. The PhyloP score for nucleotide conservation for chr9:g.14766145 is 2.55 suggesting it is under negative selection [20]. This mutation was not detected in 142 control chromosomes of Caucasian origin and 110 ethnically matched control chromosomes consistent with it not being a population polymorphism. The second instance of the p.Glu1500Val mutation was identified in patient 7 and his mother, who had craniofacial features which included hypertelorism and up-slanting palpebral fissures. Both of the proband's female siblings had also inherited the mutation; one sister had ptosis and hypertelorism (Table S2). The second individual had no abnormal craniofacial findings.

The second mutation, NM_144966.4:c.1493G>A [NP_659403.4:p.(Arg498Gln)], in exon 9, was observed in a single patient (Patient 8). Although the Grantham score is only 43, this amino acid is highly conserved with 20 vertebrate species having an arginine at this position. The PhyloP score for nucleotide conservation for chr9:g.14832559 is 2.79. It is predicted as not being tolerated by SIFT, and probably damaging by Polyphen2 [HumVar] with a score of 0.996. The proband was born to a 35 year old G1 P0 mother and 32 year old father at full term by Caesarian section. He had isolated MC corrected at six months of age. There was no family history of craniofacial dysmorphism. A fetal ultrasound at 20 weeks was reported as normal. The birth weight was 3.42 kg (75-90^th^ centile), length 50.8 cm (50-75^th^ centile). Craniosynostosis was suspected at birth and confirmed by a cranial CT scan at five months of age. The proband is currently four years of age with normal development. The mutation was proven to be paternally inherited but was absent in 138 control Caucasian chromosomes excluding a population polymorphism.

A third potential *FREM*1 mutation NM_144966.4:c.854A>G [NP_659403.4:p.(Tyr285Cys)], in exon 6, was identified in a proband in a Brazilian family however this mutation did not segregate with the disease and was detected in 1 of 142 control chromosomes of Caucasian origin and 5 of 120 ethnically matched control chromosomes. These results are consistent with this variant being a novel population polymorphism.

### Frem1 is expressed in the developing frontal and nasal sutures in mice

**Figure 2:**
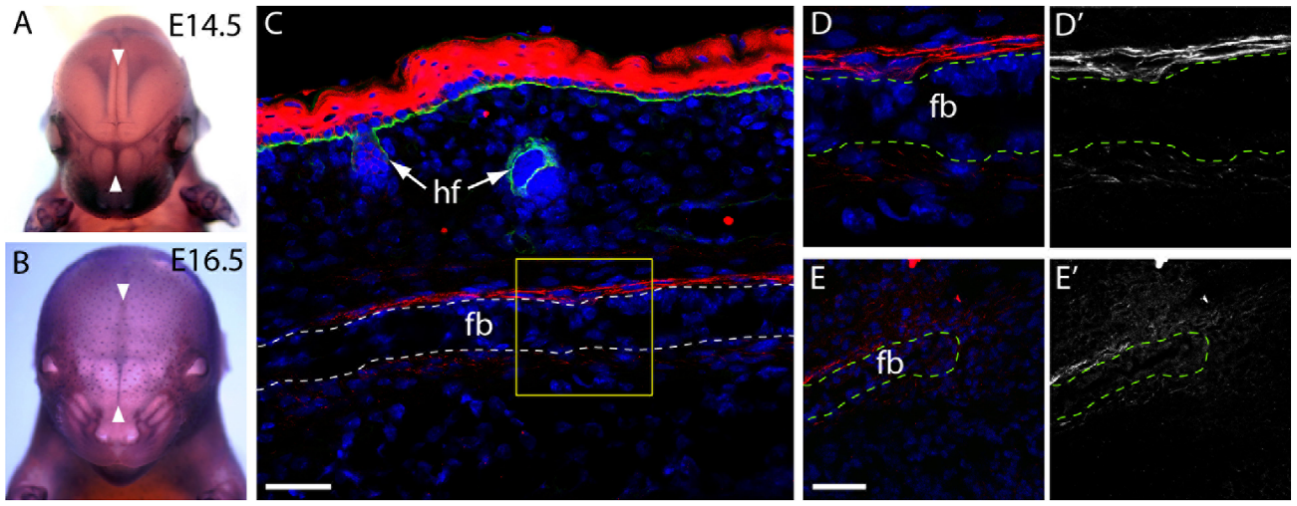
*Frem1* expression in the developing frontal and nasal sutures in mice. (a,b) *Frem1* transcripts are detected in the developing cranial sutures and in the regions fated to form the posterior-frontal suture, the metopic suture equivalent that is affected in cases of metopic craniosynostosis (white arrowheads) (c) Immunostaining for Frem1 (red) of the frontal bones at postnatal day 0 reveals expression of the protein in the pericranium and dura mater on either side of the frontal bones (dotted line; fb  =  frontal bone, hf  =  hair follicle). Samples have been counterstained with DAPI (blue) and with an antibody to entactin (green) which marks the basement membrane of the epidermis and hair follicles. The boxed area (yellow) is magnified in (d) highlighting fibrillar staining for Frem1 above and below the frontal bone (fb) (e) Frem1 protein was also noted diffusely in the suture mesenchyme at the medial edge of the frontal bones, with d' and e') showing the identical images in black and white to emphasize the fibrillar localization of Frem1. Scale bars  =  50 µm.

To ascertain a potential role for *Frem1* in the formation of the metopic suture we examined the expression of the gene prior to fusion of the suture in wildtype mice. *In situ* hybridization of mouse embryos from mid to late gestation (E14.5–E16.5) delineated expression of *Frem1* between the developing frontal bones in the region fated to form the posterior-frontal suture (Figure 2). Antibody staining at P0 highlighted fibrillar pericranial expression of Frem1 overlying the developing frontal bones as well as staining in the dura mater underlying these bones. Low levels of diffuse Frem1 staining were also noted in the osteogenic precursors between the frontal bones, further suggesting a role for the protein in the development of the posterior frontal suture.

### *Frem1* mice show craniofacial abnormalities consistent with MC

**Figure 3:**
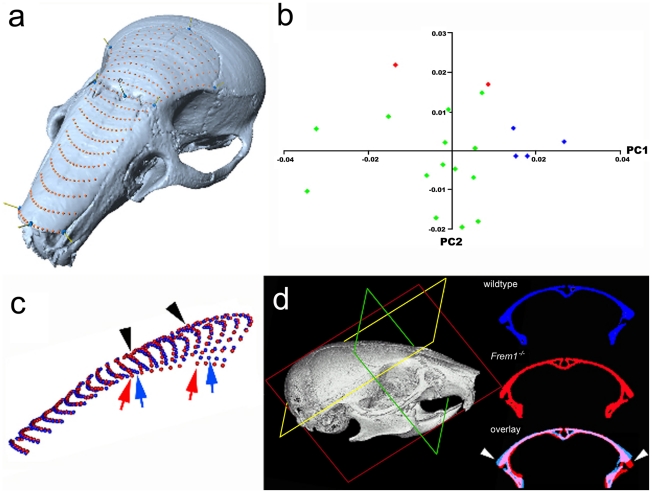
Frem1 mCT and morphometric analysis in mice are consistent with the human MC phenotype. mCT and morphometric analysis of mouse skulls at postnatal day 28 reveal anterofrontal cranial deformation (a) semi-landmark mesh over nasal and frontal bones of control skull; (b) plot of principal component 1 (PC1) and PC2 reveal that heterozygote (green dots) and homozygote (red dots) *bat* mice each have distinct craniofacial shapes from age, sex and genetic background matched controls (blue dots); (c) average mesh coordinates of control (blue dots) versus *Frem1* homozygotes (red dots) show changes in shape over the frontal bones. These differences can also be seen in (d) by comparison of identical cross-sections. These deformations are reminiscent of the trigonocephalic changes seen in patients with *FREM1* mutations.

Semi-landmark-based geometric morphometrics is a powerful means of distinguishing localized differences in form between two groups. We therefore used this approach to obtain further evidence as to whether *Frem1* mutations can cause MC. The skulls of *bat* mutant mice were imaged by mCT and analyzed using semi-landmark-based geometric morphometrics over the antero-frontal aspects of the skull (coronal suture to nasal tip). Results of principal components analysis of the posterio-frontal aspects of the cranium revealed that the first two principal components (PC1 and 2) account for more than 45% of the variation in the dataset (data not shown). PC1 is usually correlated with the size of the individuals. In geometric morphometrics, size of the specimen is determined by the centroid size, which is the square root of the summed distances between the centroid (arithmetic mean of landmarks) and each landmark of the specimen. Regression of the first two PCs against centroid size showed that size was not correlated with either of the principal components (F testpc1: 1.8 on 1 and 18 DF, p-value: 0.1964; F-testpc2: 0.9419 on 1 and 18 DF, p-value: 0.3447). In contrast, regression of the first two PCs against the groups (homozygotes, heterozygotes, and wildtype) showed a significant correlation with the genotype of the individuals (for PC1: R^2^ = 0.3943 F-statistic: 5.533 on 2 and 17 DF, p-value: 0.0141; for PC2: R^2^ = 0.3604, F-statistic: 4.789 on 2 and 17 DF, p-value: 0.02241) (Figure 3b).

Overlay of the mean positions of each semi-landmark between homozygotes and wildtype skulls showed that the homozygote upper midfaces were raised along the proximal aspect of the metopic suture (black arrowheads, Figure 3c) and were significantly wider at the level of the coronal suture (compare colored arrows, Figure 3c). Notably, the increased width in the homozygotes is more prominent posteriorly. Furthermore, the lateral aspects of the homozygote skulls decline more sharply than controls (colored arrows Figure 3c and white arrowheads in Figure 3d). Heterozygotes also show a decline at the lateral aspects although this was less pronounced than in homozygotes.

**Figure 4:**
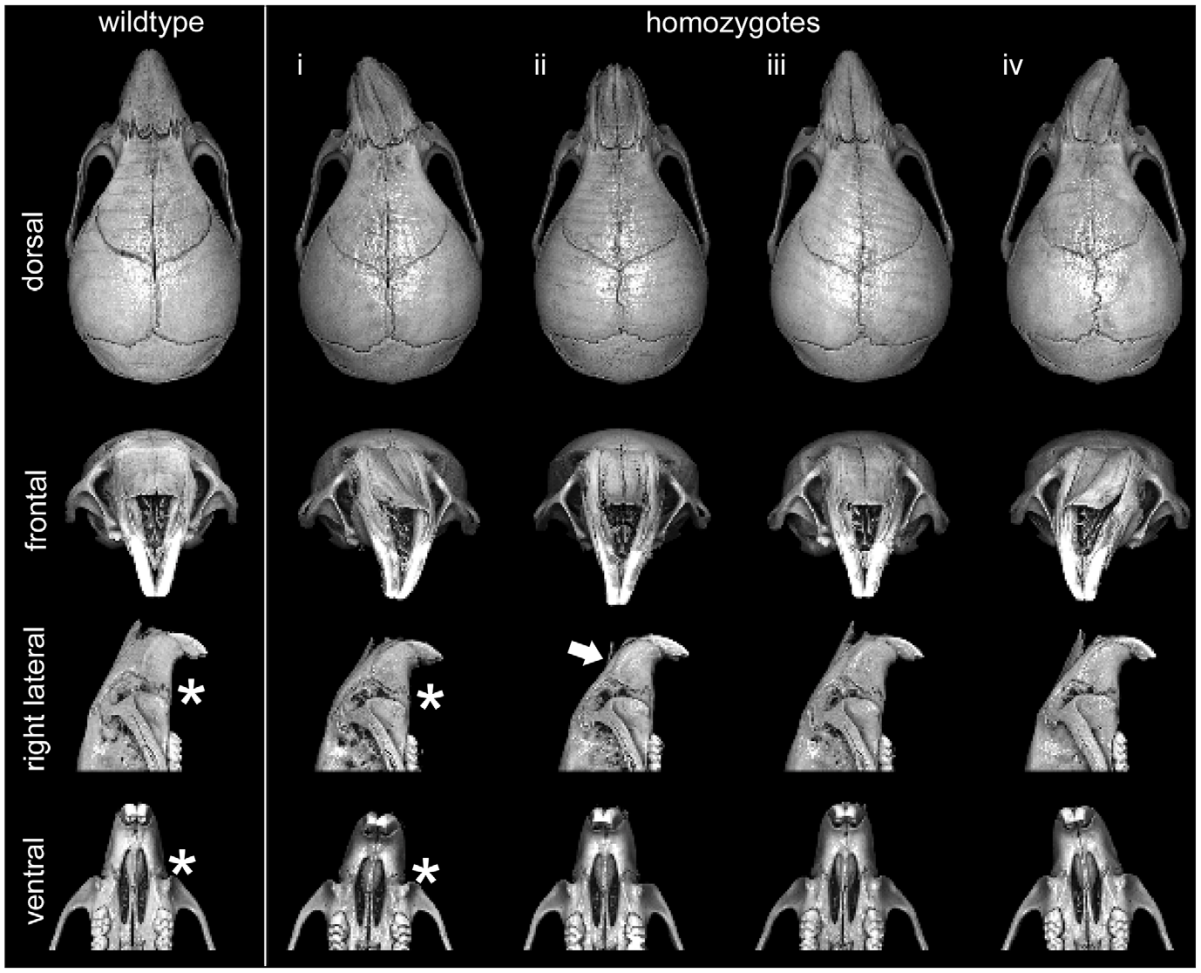
Variable presentation of midface hypoplasia/assymetry in Frem1 mutant mice. Multiple views from rendered mCT reconstructions of P28 wildtype (far left column) and homozygote male *Frem1* mice. Around 45% of homozygotes exhibited some readily apparent dysmorphology involving the midfacial region. Four examples of homozygote skull morphology observed: (i) pronounced leftward deviation of midface, (ii) uniform midface hypoplasia (shortened snout), (iii) leftward deviation extending from posterior frontal suture through to nasal tip, (iv) pronounced rightward deviation of midface. Frontal views of the homozygote skulls reveal medial depression along the internasal suture and/or marked curvature of the nasal bones (see white arrow in column (ii). Most homozygotes display abnormal maxillary-premaxillary suture morphology (compare asterisks in lateral and ventral views of control and homozygote (i).

Also of significance, one of the homozygotes from this initial set of imaged skulls showed marked deviation of the midface. To investigate this further, we scanned an additional 25 male heads from mutant and wildtype mice ranging in age from 28 days to 240 days, including 13 homozygotes and 7 heterozygotes. Visual inspection of 3D rendered images of each skull revealed varying degrees of midfacial asymmetry and/or midface hypoplasia in a significant proportion of *Frem1* homozygotes (7/16 total; 45% - when also including those used in the shape analyses; Figure 4) and to a lesser degree the *Frem1* heterozygotes (2/21 total; 9.5%). For the homozygotes, leftward deviation was more frequent than rightward deviation (5 left vs 1 right; 1 general hypoplasia). In summary, morphometric analysis of skulls from *bat* homozygous and heterozygous mice identified craniofacial malformations consistent with the craniofacial features, in particular MC and midfacial asymmetry/hypoplasia, seen in the 9p22 deletion syndrome, with the penetrance of the phenotypes in mice correlated to mutant gene dosage.

### Different *Frem1* mutant alleles show advanced fusion of the posterior frontal suture in mice

**Figure 5:**
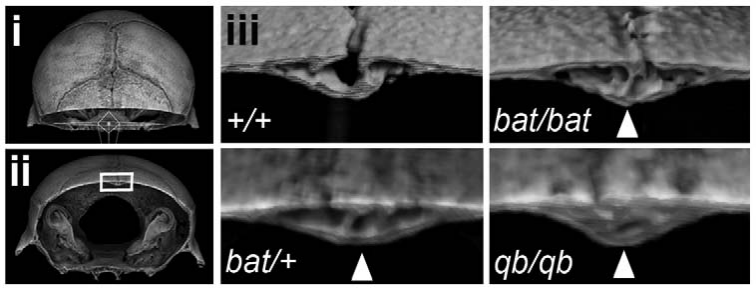
*Frem1^bat^* and *Frem1^Qbrick^* mice exhibit advanced posterior frontal suture fusion. Rendered 3D images of postnatal day 28 heads were generated from microcomputed tomographic scan data and each virtually sectioned in the coronal plane at the same position through the posterior frontal suture (i – dorsal view of position of coronal plane; ii – frontal view of rendered image in (i)). The region indicated by the rectangle is shown in (iii) for a control (+/+) skull, homozygote *Frem1^bat^ (bat/bat)* and *Frem1^Qbrick^* (qb/qb) skulls, as well as different heterozygote *Frem1^bat^* (bat/+) and *Frem1^Qbrick^* (qb/qb) skulls. The most severely affected heterozygote *Frem1^bat^* (bat/+) posterior frontal suture is also shown. Control skulls all showed sparse points of contact (typically just on the endocranial surface) between the frontal bones, indicative of the early stages of suture fusion. Fusion of the posterior frontal suture is largely completed by ∼postnatal day 45 in controls. In contrast, the *Frem1^bat/bat^* and *Frem1^qb/qb^* skulls exhibited extensive fusion both on the endocranial and ectocranial surfaces (arrowheads) at day 28, indicating advanced fusion of this suture. Heterozygotes also showed variable suture anomalies, from complete fusion to asymmetry of the suture.

To investigate the basis for the altered frontal cranial shape, three-dimensional rendered images generated from scans of *Frem1^bat^* homozygote, heterozygote and wildtype skulls were sectioned virtually in the coronal plane at the equivalent position on the posterior frontal suture. Examination of the suture in control C57BL/6J mice revealed a generally patent suture with only intermittent contact between the frontal bones either endocranially or ectocranially. In contrast, age, sex and background matched *Frem1* homozygote animals all exhibited complete fusion of the PF suture (Figure 5). Notably, 42% of heterozygous animals showed variable sutural anomalies including complete fusion (1/14; 7%), obvious sutural asymmetry (3/14; 21%) and advanced endocranial suture fusion (2/14; 14%). Importantly, the findings in the *Frem1^bat^* mice were validated by the analysis of a litter of mice carrying a second *Frem1* mutation, the *Frem1^Qbrick^* null allele; the single homozygote mouse showed complete fusion of the PF suture, while two of the three heterozygotes analyzed showed similar suture anomalies to those seen in *Frem1^bat^* heterozygotes (Figure 5).

To investigate whether either of the two remaining genes within the refined critical interval might also contribute to the craniofacial phenotype of the 9p22 deletion syndrome, we first analyzed a small number of *Zdhhc21^dep/dep^* homozygotes. Comparison of these mice to the analyzed *Frem1* mice (37d vs 28-30d), demonstrated that none had PF suture fusion or sutural anomalies (data not shown). Assessment of the cranioskeletal phenotype of late stage homozygous *Nfib^-/-^* fetuses with age matched C57BL/6J control embryos showed that the *Nfib^-/-^* fetuses exhibited reduced overall size and relatively delayed cranial bone growth (data not shown), but without evidence of premature suture fusion.

## Discussion

In this study we report that *FREM1* is a dominantly-acting metopic craniosynostosis gene based upon findings of: i) patients with trigonocephaly with CNVs which disrupt or delete the coding sequence of the gene; ii) missense mutations in *FREM1* in patients with isolated MC; iii) expression of mouse Frem1 gene and protein in the posterior frontal suture (metopic suture equivalent); iv) advanced fusion of the PF suture, altered frontal bone curvature and midfacial malformations in two independent mouse lines carrying distinct *Frem1* loss-of-function mutations, and: v) absence of an overt cranial suture phenotype in mice deficient in either of the two remaining genes (*Zdhhc21* and *Nfib*) that reside within the 9p22 critical interval.

FREM1 is composed of several functional domains, including a conserved signal sequence, a CALXb calcium-binding loop typically found in Na^+^-Ca^+^ exchange proteins, 12 chondroitin sulfate proteoglycan (CSPG) elements, and a C-terminal lectin Type C domain [21]. FREM1 is a basement membrane protein which forms a ternary complex with the FRAS1 and FREM2 proteins, although the topology and stoichiometry of the subunits is not certain. *FREM1* orthologs are highly conserved throughout evolution and the 12 CSPG repeats are highly homologous with each other. Both point mutations observed in the MC cohort affect residues within the CSPG repeats whose physico-chemical properties are also strongly conserved in evolutionary history. Prediction of the consequences of these mutations, based on similarity to the structurally related NG2 protein, suggest they affect surface residues of FREM1, potentially disturbing the interactions with other proteins. CSPG repeats of the NG2 proteoglycan have been shown to interact with basic fibroblast growth factor [22]. It is intriguing to speculate that FREM1 could also bind FGFs and that the craniosynostosis associated with a mutation in these repeats arises through altered FGFR activation as a result of increased intrasutural FGF availability or its affinity for their receptors. A similar mechanism has recently been shown for the *Eks* mouse which carries a gain-of-function mutation in Fgf9 [14].

Recently, Alazami et al. (2009) demonstrated expression of *Frem1* in the developing midface by whole mount *in situ* hybridization in normal embryonic day 11.5 mice, consistent with the midfacial phenotypes seen in both BNAR and FREM1-trigonocephaly [15]. We have now demonstrated that expression of *Frem1* mRNA is also present in the developing interfrontal suture. These findings are also validated by immunohistochemical detection of Frem1 protein within the sutural mesenchyme as well as in the dura mater and pericranium prior to the initiation of the posterior frontal suture at postnatal day 7. We speculate that expression of FREM1 within the intrasutural mesenchyme would be consistent with a role in modulating Fgf or other growth factor availability.

An important role for Frem1 in regulating suture patency is supported by our mCT investigations of *Frem1^bat^* and *Frem1^Qbrick^* mice. Previous studies have established that the posterior frontal suture in C57BL/6J mice begins to fuse after postnatal day 25 and morphologically completes fusion around day 45. This timing is consistent with the findings in our postnatal day 28-30 wildtype mice, which show only sparse contact between the frontal bones. In contrast, virtual cross-sectional analysis of *Frem1^bat^* and *Frem1^Qbrick^* homozygotes revealed almost complete fusion of the posterior frontal suture, both ectocranially and endocranially (Figure 5). Importantly, heterozygotes of each mutation also showed variable posterior frontal sutural anomalies ranging from complete fusion to atypical sutural asymmetry to a normal appearing suture. Furthermore geometric morphometric analysis of heterozygote and homozygote *Frem1^bat^* skulls and wildtype genetic background controls revealed that *Frem1* mutant mice show altered frontal bone curvature, in particular at the level of the posterior frontal suture. Although more evident in the homozygote skulls, the medial aspects over the posterior frontal suture appear raised relative to the lateral aspects which decline more sharply than in controls (see Figure 3a and 3b). The variably penetrant posterior frontal suture anomalies in *Frem1* heterozygotes and the lack of overt cranial and/or sutural defects in homozygous *Zdhhc21* and *Nfib* mutants supports a causative role for *FREM1* haploinsufficiency in the 9p22.3-trigonocephaly in humans. Further to this, the analyses of the mouse craniofacial skeleton also revealed that homozygous *Frem1^bat^* mice, and to a lesser extent heterozygote mice, generally have a shorter upper midface, including nasal bone and premaxilla (midfacial hypoplasia/asymmetry) and slightly wider nasal bones (Figure 4). These findings are consistent with the earlier reported expression of *Frem1* in the developing facial prominences and the role of recessive *FREM1* mutations in BNAR syndrome [15]. Notably, midface hypoplasia is one of the major clinical features of 9p22.3-trigonocephaly and was noted in at least six of the eight probands reported here (Table 1).

The *FREM1* mutational classes identified in humans include structural variants that interrupt the gene, CNVs of the entire *FREM1* locus and point mutations of the *FREM1* coding sequence. The observed mutational heterogeneity is reminiscent of other dominantly-acting mutations of ECM multimeric proteins such as collagen type 1 α1, collagen type 1 α2 [23] and fibrillin [24] where multi-exon deletions and missense mutations both result in the same disease phenotype. It is interesting to note however, that the *FREM1* mutations identified in this study are heterozygous (dominantly acting) with the morphological consequences largely restricted to the craniofacial complex (metopic suture and midface) compared with the multi-organ involvement in the autosomal recessive disorder, BNAR. The phenomenon of dominant and recessive mutations in the same gene is well described and associated with a range of molecular etiologies. Classically, recessive mutations may produce a spectrum of disorder severity based on allele dosage, as has been described in familial hypercholesterolemia [25]. Alternatively autosomal dominant gain of function mutations may be associated with a different spectrum of disease compared with loss of function mutations. This is exemplified by heterozygous gain of function mutations in *RET* which are associated with thyroid carcinoma and multiple endocrine neoplasia types 2A and 2B versus homozygous loss of function mutations in Hirschsprung disease [26]. Finally, dominant and recessive mutations may involve different stages of protein biosynthesis or functional domains, as described for mutations in the insulin gene in which dominant neonatal diabetes arises from mutations associated with misfolding of proinsulin whereas recessive mutations reduce insulin biosynthesis [27]. The difference in range and severity of organ involvement is consistent with MC arising as a result of haploinsufficiency for *FREM1*. BNAR and FREM1-trigonocephaly could therefore be considered to form a spectrum of disorders within the same genetic pathway distinguished by dominant and recessive inheritance.

Phenotypic variability and incomplete penetrance are typical findings in the *TWIST1* and *FGFR*-associated craniosynostoses and are therefore not unexpected findings in this MC patient cohort. The p.Glu1500Val mutation was present in *de novo* form in one affected individual. In a second family this mutation was inherited from the proband's mother who has facial features of hypertelorism (>3SD) and upslanting palpebral fissures. The mutation was also present in three siblings, one of whom (the proband) had trigonocephaly and microcephaly (<3SD), a sister who had ptosis and hypertelorism, and a third sibling who has relative hypertelorism but no significant findings. Similarly, the p.Arg498Gln mutation was inherited from a clinically unaffected father.

In line with the variable clinical presentations, *Frem1^bat^* mice have been previously reported as showing a number of variably penetrant features including renal agenesis (∼20%), limb (digit) anomalies (<5%) and unilateral or bilateral crytophthalmos [21]. However, mouse lines carrying other mutant *Frem1* alleles (that arose on different inbred backgrounds) typically do not present with renal phenotypes and show variable penetrance of other features as well [28], [29]. The variable expression and penetrance of skull deformations in the inbred *Frem1^bat^* line is consistent with these observations. The influence of genetic background on the presentation of the Frem1 mutant phenotype and that of the broader family of blebs mouse mutants which carry mutations in the other Fras/Frem genes has been confirmed by placing the same mutations on different backgrounds (I. Smyth, personal communication). We therefore speculate that the variable penetrance and expressivity of the human *FREM1* mutations is due to a combination of factors, including genetic modifiers and epigenetic modulators. Such penetrance and expressivity must therefore be considered when counseling patients in regard to recurrence risk. This group of clinical findings suggest that the phenotypic consequences of *FREM1* mutations in humans and mice include but are not limited to MC.

In summary, we provide evidence that *FREM1* mutations are associated with trigonocephaly. Taken together, our data support a role for *FREM1* in the closure of the metopic as well as premaxillary-maxillary sutures and suggest further avenues for study into unisutural synostosis biology. Furthermore, we present the *Frem1* mouse as a new animal model for both trigonocephaly and facial asymmetry.

## Materials and Methods

### Ethics statement

All animals were handled in strict accordance with good animal practice as defined by the relevant national and/or local animal welfare bodies, and all animal work was approved by the University of Washington Research Ethics Committee.

### Patients

109 patients with non-syndromic MC were ascertained from five participating centers of the International Craniosynostosis Consortium (http://genetics.ucdavis.edu/icc.cfm) (University of California, Davis, USA n = 34; Seattle Children's Hospital, Seattle, USA, n = 33; Universidade de São Paulo, São Paulo, Brazil, n = 24; Australian Craniofacial Unit, Adelaide, Australia, n = 14; and RUNMC, Nijmegen, The Netherlands n = 4). DNA was isolated or purified using a QIAamp DNA mini kit (QIAgen, the Netherlands) following standard procedures. Parental samples were collected when available, and informed consent was obtained in all cases following local institutional human research ethics guidelines. Mutation pre-screening of the regions of the *FGFR* and *TWIST1* genes associated with syndromic craniosynostosis was performed by bi-directional DNA sequence analysis; including *FGFR1* exon 7, *FGFR2* exons 3, 5, 8, 10, 11 and 14-17, *FGFR3* exons 7 and 10 and *TWIST1* [30], [31].

### Fine mapping of CNVs using chromosome 9 microarray analyses

Chromosome 9 specific oligonucleotide microarray analyses were performed using a 385K array following the manufacturer's instructions (Roche NimbleGen). The acquired images were analyzed using NimbleScan V2.4 extraction software (Roche NimbleGen). Data were visualized in SignalMap V1.9 software to determine the boundaries of the CNVs (Roche NimbleGen).

### Multiplex ligation-dependent probe amplification (MLPA) of *FREM1*

An in-house MLPA kit was developed to screen for CNVs at 9p22.3. A set of uniquely-sized MLPA probes hybridizing to exon 2 of *CER1*, exons 2, 9, 16, 23, 31 of *FREM1* and to intron 8 of *TTC39B* were developed. These MLPA probes were combined with four unlinked standard control probes. All probes were designed following the protocol of MRC-Holland and the sequences are provided in Table S3 (http://www.mlpa.com/index.htm). Hybridization, ligation and amplification of the MLPA probes were performed as described previously [32]. Amplification products were identified and quantified by capillary electrophoresis on an ABI3730 or 3100 Genetic Analyzer, using GeneMapper software (Applied Biosystems).

### Sequence analysis of *CER1* and *FREM1*

All coding regions and adjacent splice sites of *CER1* (NM_005454) and *FREM1* (NM_144966) were amplified and sequenced (PCR primers and conditions available on request). PCR products were cycle sequenced (ABI 3730 or 3100) and analyzed for mutations using the Vector NTI v11 software together with visual inspection. Potential mutations were tested for *de novo* occurrence through mutation analysis of parental DNA where available. Sequence variants are described following the HGVS nomenclature guidelines.

### Mouse strains

Mice carrying the ENU-generated *Frem1^bat^* mutation maintained on a C57Bl/6J background were used for this study. The *bat* allele is the result of a single nucleotide change that affects the normal mRNA splicing of exon 25 of the mouse *Frem1* gene, leading to a frameshift and premature truncation of the protein. The mutant allele is thought to be hypomorphic rather than a null allele [21]. Standard mating protocols were used to generate heterozygote and homozygote animals that were each identified by PCR-based genotyping [21]. Mice were sexed based on genital morphology at weaning. A single litter of mice carrying a *Frem1* null allele (*Frem1^Qbrick^*) was also made available for analysis, including three heterozygous animals, one surviving homozygote and three littermate C57BL/6J controls. These mice were generated by a traditional gene targeting approach [28] and also maintained on a pure C57BL/6J background. A small number of mice from mutant lines representing both of the remaining genes that reside within the refined critical interval, *ZDHHC21* and *NFIB*, were also obtained. The *Zdhhc21^dep^* line [33] carries a three base pair deletion (single amino acid deletion) that results in protein mislocalization and loss of enzyme activity and is associated with delayed hair shaft differentiation. The remaining mutant carries a null allele of *Nfib*, generated through traditional gene targeting [34]. *Nfib* heterozygotes are apparently healthy and show no overt phenotype, while *Nfib* homozygotes die just prior to birth. A limited number of late stage homozygous *Nfib^-/-^* fetuses were available. Both lines were provided on a C57BL/6 background.

### Micro-computed tomography (mCT) and skull shape analysis

For the purpose of assessing suture patency and comparing cranial shape, a total of 21 specimens were subjected to mCT using a Skyscan model 1076 in-vivo microtomograph (Skyscan, Belgium). All specimens were scanned at the Small Animal Tomographic Analysis (SANTA) Facility at the University of Washington, using and following optimized protocols (0.018 mm voxel resolution, 65 kV, 150 uA, 1.0 mm Al filter) previously reported [35], [36]. Out of the 21 specimens, three were homozygotes, four were wild-type and the remainder were *Frem1^bat^* heterozygotes. A further seven 4 week-old *Frem1^Qbrick^* mice (three heterozygotes, one homozygote, and three C57Bl/6J controls) were also scanned. Raw scan data were reconstructed using NRecon software (Skyscan, Belgium) and rendered using the volume exploration software, Drishti V2.0 (http://anusf.anu.edu.au/Vizlab/drishti/). Virtual cuts in the coronal plane were then made at the same planar level in each rendered skull to assess the patency of the posterior frontal suture. To ensure direct comparability, all scan, reconstruction and rendering parameters were kept identical for all skulls.

3D mesh models of each skull created using the CTan software (Skyscan, Belgium) and were then imported into Rapidform (Inus Technology) in which the mandibles and other unwanted elements were segmented out. Cleaned meshes were then imported into the 3D landmark software developed by the Institute for Data Analysis and Visualization (IDAV Landmark). A flexible patch was applied to the region between the rostrum and the coronal suture in the dorsal view. Each patch had 9 control points anchored to biologically homologous structures to ensure the consistency of the patch placement (Figure 3a inset). Finally, the patch was converted into a 15×21 deformable grid, and the 3D coordinates of 315 grid points were exported. Due to some mesh inconsistencies, the Landmark software could place 309 of 315 grid point in all skulls, and these were used in all subsequent analyses. 3D renderings of the skulls indicated a high level of deformation in one of the initial homozygous specimens. Although biologically highly relevant, this specimen was excluded from the shape analyses to preclude any undue influence of this outlier.

Generalized procrustes analysis (GPA) was used to quantify and visualize the localized shape differences among the groups as implemented in the R “shapes” package [37]. The bones were ordinated in shape-space using principal components analysis. Each principal component summarizes a unique portion of the shape variation that is not explained by any of the preceding components. Typically, the morphospace is centered on the mean (or consensus) shape configuration of the GPA, so that the each point in the morphospace can be visualized as deviation from the mean shape. Principal components also can be used as shape variables on which statistical tests can be applied.

Subsequent to the detailed shape analyses, a further twenty five *Frem1^bat^* males (thirteen homozygotes, seven heterozygotes, and five controls) ranging from postnatal day 28 through to postnatal day 240 were scanned and 3D rendered models generated using Drishti V2.0 (http://sf.anu.edu.au/Vizlab/drishti/). Five 37 day old *Zdhhc21^dep/dep^* mice were also obtained for imaging. In addition, three embryonic day 18.5 *Nfib^-/-^* mice were analysed. All skulls were visually inspected and any gross malformations recorded.

### Analysis of Frem1 expression

Frem1 transcripts were detected using specific riboprobes to the 3′ UTR of the gene as described previously [21]. A rabbit polyclonal Frem1 antisera was raised against Frem1 CSPG domain 11 and partial domain 12 (aa 1500–1637) expressed and purified as a His-tagged recombinant protein [38]. Immunohistochemistry was performed on 10 micrometer frozen sections of P0 mouse heads using antibodies to Frem1 (above) and entactin (Abcam) detected using Alexa conjugated secondary antibodies (Molecular Probes). Images were captured on a Leica SP5 confocal microscope.

## Supporting Information

Table S1 Syndromes with metopic synostosis/trigonocephaly.
(DOC)

Table S2 Craniofacial measurements.
(DOC)

Table S3 MLPA probes used for CNV screening of *FREM1.*
(DOC)
