# Supplementary material for: Heterozygous Mutations of FREM1 Are Associated with an Increased Risk of Isolated Metopic Craniosynostosis in Humans and Mice
Source: PLoS Genet. 2011 Sep 8;7(9):e1002278. doi: 10.1371/journal.pgen.1002278 (PMC3169541; doi:10.1371/journal.pgen.1002278)
Supplement: Table S1 — Syndromes with metopic synostosis/trigonocephaly. (DOC) [file pgen.1002278.s001.doc]

**Table S1**

Syndromes with metopic synostosis / trigonocephaly

|  | **OMIM** | **Cytogenetic Band** | **Gene** |
| --- | --- | --- | --- |
| ***CRANIOFACIAL SYNDROMES*** |  |  |  |
| *BALLER-GEROLD SYNDROME; BGS* | #218600 | 8q24.3 |  |
| *C SYNDROME* | #211750 | 3q13.13 | *CD96 ANTIGEN; CD96* |
| *C-LIKE SYNDROME* | #605039 *606037 | 3q13.13 | *CD96 ANTIGEN; CD96* |
| *CLEIDOCRANIAL DYSPLASIA; CCD* | #119600 | 6p21 |  |
| *CRANIOSYNOSTOSIS-MENTAL RETARDATION SYNDROME OF LIN AND GETTIG* | 218649 |  |  |
| *FIBROBLAST GROWTH FACTOR RECEPTOR 1, FGFR1* | *136350 | 8p11.2-p11.1 | *FGFR1* |
| *FIBROBLAST GROWTH FACTOR RECEPTOR 2; FGFR2* | *176943 | 10q26 | *FGFR2* |
| *FIBROBLAST GROWTH FACTOR RECEPTOR 3, MUENKE SYNDROME* | #602849 | 4p16.3 | *FGFR3* |
| *OPITZ GBBB SYNDROME, AUTOSOMAL DOMINANT* | #145410 | 22q11.2 |  |
| *PARIETAL FORAMINA; PFM* | #168500 | 5q34-q35 11p11.2 | *MSX2 ALX4* |
| *SAETHRE-CHOTZEN SYNDROME; SCS* | #101400 | 10q26, 7p21 | *TWIST1* |
| *TRIGONOCEPHALY WITH SHORT STATURE AND DEVELOPMENTAL DELAY* | 314320 |  |  |
| *TRIGONOCEPHALY, NONSYNDROMIC* | #190440 | 8p11.2-p11.1 | *FGFR1?* |
|  |  |  |  |
| ***CHROMOSOMAL IMBALANCES*** |  |  |  |
| *CHROMOSOME 1p36 (atypical proximal) DELETION SYNDROME* | [#607872](http://www.ncbi.nlm.nih.gov/entrez/dispomim.cgi?cmd=entry&id=607872) | [1p36](http://www.ncbi.nlm.nih.gov/Omim/getmap.cgi?l607872) |  |
| *CHROMOSOME 1q43-q44 DELETION SYNDROME* | #612337 | 1q42-q44 |  |
| *CHROMOSOME 2p15-p16 DELETION SYNDROME* | [#612513](http://www.ncbi.nlm.nih.gov/entrez/dispomim.cgi?cmd=entry&id=612513) | [2p16.1-p15](http://www.ncbi.nlm.nih.gov/Omim/getmap.cgi?l612513) |  |
| *WILLIAMS-BEUREN REGION 7q11.23 DELETION SYNDROME* | [#609757](http://www.ncbi.nlm.nih.gov/entrez/dispomim.cgi?id=609757) | [7q11.23](http://www.ncbi.nlm.nih.gov/Omim/getmap.cgi?l609757) |  |
| *CHROMOSOME 7q11.23 DUPLICATION SYNDROME* | [#609757](http://www.ncbi.nlm.nih.gov/entrez/dispomim.cgi?cmd=entry&id=609757) | [7q11.23](http://www.ncbi.nlm.nih.gov/Omim/getmap.cgi?l609757) |  |
| *CHROMOSOME 8p12-p21 DELETION SYNDROME* |  | 8p12-p21 |  |
| *CHROMOSOME 9p DELETION SYNDROME* | [#158170](http://www.ncbi.nlm.nih.gov/entrez/dispomim.cgi?id=158170) | [9p](http://www.ncbi.nlm.nih.gov/Omim/getmap.cgi?l158170) |  |
| *HASPESLAGH SYNDROME, 9p DEL/6q DUP PTERYGIA, MENTAL*  *RETARDATION, AND DISTINCTIVE CRANIOFACIAL FEATURES* | [#177980](http://www.ncbi.nlm.nih.gov/entrez/dispomim.cgi?id=177980) | 9p/6q unbalanced translocation |  |
| *JACOBSEN SYNDROME, CHROMOSOME 11q23 DELETION SYNDROME,*  *THROMBOCYTOPENIA, PARIS-TROUSSEAU TYPE; TCPT* | [#188025 #147791](http://www.ncbi.nlm.nih.gov/entrez/dispomim.cgi?id=188025) | [11q23](http://www.ncbi.nlm.nih.gov/Omim/getmap.cgi?l188025) |  |
| *CHROMOSOME 14q12 DELETION SYNDROME* |  | 14q12 |  |
| *CHROMOSOME 17q21.32 DELETION SYNDROME* |  | 17q21.32 |  |
| *CHROMOSOME 20q13 DELETION SYNDROME* |  | 20q13 |  |
| *CHROMOSOME 22q11.2 DELETION SYNDROME* | [#611867](http://www.ncbi.nlm.nih.gov/entrez/dispomim.cgi?cmd=entry&id=611867) | [22q11.2](http://www.ncbi.nlm.nih.gov/Omim/getmap.cgi?l611867) |  |
| *CHROMOSOME Xq28 DUPLICATION SYNDROME* |  | Xq28 |  |
|  |  |  |  |
| ***KNOWN SYNDROMES*** |  |  |  |
| *BECKWITH-WIEDEMANN SYNDROME; BWS* | [#130650](http://www.ncbi.nlm.nih.gov/entrez/dispomim.cgi?id=130650) | [11p15.5 5q35](http://www.ncbi.nlm.nih.gov/Omim/getmap.cgi?l130650) |  |
| *CEREBRO-OCULO-FACIO-SKELETAL SYNDROME (PENA-SHOKEIR Type 2)* | [#214150](http://www.ncbi.nlm.nih.gov/entrez/dispomim.cgi?cmd=entry&id=214150) | [10q11](http://www.ncbi.nlm.nih.gov/Omim/getmap.cgi?l214150) | *ERCC6* |
| *COFFIN-SIRIS, AR inheritance* | [135900%](http://www.ncbi.nlm.nih.gov/entrez/dispomim.cgi?cmd=entry&id=135900) |  |  |
| *DONNAI-BARROW SYNDROME* | [#222448](http://www.ncbi.nlm.nih.gov/entrez/dispomim.cgi?id=222448) | [2q24-q31](http://www.ncbi.nlm.nih.gov/Omim/getmap.cgi?l222448) |  |
| *EHLERS-DANLOS SYNDROME, TYPE VII, AUTOSOMAL RECESSIVE* | [#225410](http://www.ncbi.nlm.nih.gov/entrez/dispomim.cgi?id=225410) | [5q23](http://www.ncbi.nlm.nih.gov/Omim/getmap.cgi?l225410) |  |
| *FG SYNDROME 5; FGS5* | 300581% | [Xq22.3](http://www.ncbi.nlm.nih.gov/Omim/getmap.cgi?l300581) |  |
| *FLOATING-HARBOR SYNDROME* | [136140](http://www.ncbi.nlm.nih.gov/entrez/dispomim.cgi?id=136140) |  |  |
| *FRONTOOCULAR SYNDROME* | [605321](http://www.ncbi.nlm.nih.gov/entrez/dispomim.cgi?id=605321) |  |  |
| *FRYNS-AFTIMOS SYNDROME* | [606155](http://www.ncbi.nlm.nih.gov/entrez/dispomim.cgi?id=606155) |  |  |
| *IRIS COLOBOMA WITH PTOSIS, HYPERTELORISM, AND MENTAL*  *RETARDATION (BARAITSER-WINTER)* | [243310](http://www.ncbi.nlm.nih.gov/entrez/dispomim.cgi?id=243310) |  |  |
| *LACRIMOAURICULODENTODIGITAL SYNDROME; LADD* | [#149730](http://www.ncbi.nlm.nih.gov/entrez/dispomim.cgi?id=149730) | [5p13-p12, 4p16.3](http://www.ncbi.nlm.nih.gov/Omim/getmap.cgi?l149730) |  |
| *OROFACIODIGITAL SYNDROME VI; OFD6* | [277170%](http://www.ncbi.nlm.nih.gov/entrez/dispomim.cgi?id=277170) |  |  |
| *POTOCKI-LUPSKI SYNDROME; PTLS* | [#610883](http://www.ncbi.nlm.nih.gov/entrez/dispomim.cgi?id=610883) | [17p11.2](http://www.ncbi.nlm.nih.gov/Omim/getmap.cgi?l610883) |  |
| *SCHINZEL-GIEDION MIDFACE-RETRACTION SYNDROME* | [269150%](http://www.ncbi.nlm.nih.gov/entrez/dispomim.cgi?id=269150) |  |  |
|  |  |  |  |
| ***RARER SYNDROMES*** |  |  |  |
| *AGLOSSIA SITUS INVERVSUS, uncertain inheritance* | [612776%](http://www.ncbi.nlm.nih.gov/entrez/dispomim.cgi?cmd=entry&id=612776) |  |  |
| *AL-SANNAA SYNDROME TRIGONOMICROCEPHALY-ASD-SYNDACTYLY-CAFE-*  *AU-LAIT SPOTS, uncertain inheritance* |  |  |  |
| *ARTHROGRYPOSIS, DISTAL, WITH MENTAL RETARDATION AND*  *CHARACTERISTIC FACIES* | [208081](http://www.ncbi.nlm.nih.gov/entrez/dispomim.cgi?id=208081) |  |  |
| *BLEPHAROPHIMOSIS WITH FACIAL AND GENITAL ANOMALIES AND MENTAL*  *RETARDATION* | [604314](http://www.ncbi.nlm.nih.gov/entrez/dispomim.cgi?id=604314) |  |  |
| *BROSNAN SYNDROME, XY GONADAL DYSGENESIS PLUS OTHER ANOMALIES,*  *AR inheritance* | [233430](http://www.ncbi.nlm.nih.gov/entrez/dispomim.cgi?cmd=entry&id=233430) |  |  |
| *CHITAYAT SYNDROME, MR, FACIAL ANOMALIES, HYPOPITUITARISM AND*  *ARTHROGRYPOSIS, autosomal recessive* | [208080](http://www.ncbi.nlm.nih.gov/entrez/dispomim.cgi?cmd=entry&id=208080) |  |  |
| *CRISTIAN SYNDROME, MENTAL RETARDATION, SKELETAL DYSPLASIA, AND*  *ABDUCENS PALSY; MRSD* | [309620%](http://www.ncbi.nlm.nih.gov/entrez/dispomim.cgi?id=309620) | [Xq28](http://www.ncbi.nlm.nih.gov/Omim/getmap.cgi?l309620) |  |
| *FINE-LUBINSKI SYNDROME, BRACHYCEPHALY, DEAFNESS, CATARACT,*  *MICROSTOMIA AND MR,uncertain inheritance* | [601353](http://www.ncbi.nlm.nih.gov/entrez/dispomim.cgi?cmd=entry&id=601353) |  |  |
| *GARCIA LURIE SYNDROME, ATELENCEPHALY-APROSENCEPHALY,*  *uncertain/AR inheritance* | [207770](http://www.ncbi.nlm.nih.gov/entrez/dispomim.cgi?cmd=entry&id=207770) |  |  |
| *GROWTH AND MENTAL RETARDATION, MANDIBULOFACIAL DYSOSTOSIS,*  *MICROCEPHALY, AND CLEFT PALATE* | [610536](http://www.ncbi.nlm.nih.gov/entrez/dispomim.cgi?id=610536) |  |  |
| *HYPOSPADIAS-MENTAL RETARDATION SYNDROME* | [241760](http://www.ncbi.nlm.nih.gov/entrez/dispomim.cgi?id=241760) |  |  |
| *LEHMAN LATERAL MENINGOCELE SYNDROME* | [130720%](http://www.ncbi.nlm.nih.gov/entrez/dispomim.cgi?id=130720) |  |  |
| *LENZ-MAJEWSKI HYPEROSTOTIC DWARFISM* | [151050](http://www.ncbi.nlm.nih.gov/entrez/dispomim.cgi?id=151050) |  |  |
| *MICROCEPHALY WITH CHEMOTACTIC DEFECT AND TRANSIENT*  *HYPOGAMMAGLOBULINEMIA, XLR* | [251240](http://www.ncbi.nlm.nih.gov/entrez/dispomim.cgi?id=251240) |  |  |
| *PIEBALD TRAIT; PBT* | [#172800](http://www.ncbi.nlm.nih.gov/entrez/dispomim.cgi?id=172800) | [8q11](http://www.ncbi.nlm.nih.gov/Omim/getmap.cgi?l172800) | *KIT* |
| *PORENCEPHALY, CEREBELLAR HYPOPLASIA AND INTERNAL*  *MALFORMATIONS, AR inheritance* | [601322](http://www.ncbi.nlm.nih.gov/entrez/dispomim.cgi?cmd=entry&id=601322) |  |  |
| *RENPENNING SYNDROME 1; RENS1* | [#309500](http://www.ncbi.nlm.nih.gov/entrez/dispomim.cgi?id=309500) | [Xp11.23](http://www.ncbi.nlm.nih.gov/Omim/getmap.cgi?l309500) | *PQBP1* |
| *SCHILBACH-ROTT SYNDROME OCULAR HYPOTELORISM, SUBMUCOSAL*  *CLEFT PALATE, AND HYPOSPADIAS* | [164220%](http://www.ncbi.nlm.nih.gov/entrez/dispomim.cgi?id=164220) |  |  |
| *SERPENTINE FIBULA-POLYCYSTIC KIDNEY SYNDROME* | [600330](http://www.ncbi.nlm.nih.gov/entrez/dispomim.cgi?id=600330) |  |  |
| *TRIGONOBRACHYCEPHALY, BULBOUS BIFID NOSE, MICROGNATHIA, AND*  *ABNORMALITIES OF THE HANDS AND FEET* | [275595](http://www.ncbi.nlm.nih.gov/entrez/dispomim.cgi?id=275595) |  |  |
|  |  |  |  |
| ***SYNDROMES WITH METABOLIC/CELLULAR/TERATOGENIC DEFECTS*** |  |  |  |
| *ADENYLOSUCCINASE DEFICIENCY* | [#103050](http://www.ncbi.nlm.nih.gov/entrez/dispomim.cgi?id=103050) | [22q13.1](http://www.ncbi.nlm.nih.gov/Omim/getmap.cgi?l103050) | *ADSL* |
| *CDG TYPE Ie* | [#608799](http://www.ncbi.nlm.nih.gov/entrez/dispomim.cgi?cmd=entry&id=608799) | [20q13.13](http://www.ncbi.nlm.nih.gov/Omim/getmap.cgi?l608799) | *DPM1* |
| *DEVELOPMENTAL DELAY, EPILEPSY AND NEONATAL DIABETES (DEND)* | [#606176](http://www.ncbi.nlm.nih.gov/entrez/dispomim.cgi?cmd=entry&id=606176) | [11p15.1](http://www.ncbi.nlm.nih.gov/Omim/getmap.cgi?l600937) | *KCNJ11* |
| *EXCISION-REPAIR CROSS-COMPLEMENTING, GROUP 6; ERCC6, MACULAR*  *DEGENERATION, ARMD5* | [609413](http://www.ncbi.nlm.nih.gov/entrez/dispomim.cgi?id=609413) | [10q11](http://www.ncbi.nlm.nih.gov/Omim/getmap.cgi?l609413) | *ERCC6* |
| *HURLER SYNDROME* | [#607014](http://www.ncbi.nlm.nih.gov/entrez/dispomim.cgi?id=607014) | [4p16.3](http://www.ncbi.nlm.nih.gov/Omim/getmap.cgi?l607014) |  |
| *ORNITHINE TRANSCARBAMYLASE DEFICIENCY, HYPERAMMONEMIA DUE*  *TO, VALPROATE SENSITIVITY, INCLUDED* | [#311250](http://www.ncbi.nlm.nih.gov/entrez/dispomim.cgi?id=311250) | [Xp21.1](http://www.ncbi.nlm.nih.gov/Omim/getmap.cgi?l311250) | OTC |
| *PYRUVATE DEHYDROGENASE E3-BINDING PROTEIN DEFICIENCY* | [#245349](http://www.ncbi.nlm.nih.gov/entrez/dispomim.cgi?id=245349) | [11p13](http://www.ncbi.nlm.nih.gov/Omim/getmap.cgi?l245349) | PDHX |
| *VALPROATE EMBRYOPATHY, SUSCEPTIBILITY TO* | [609442](http://www.ncbi.nlm.nih.gov/entrez/dispomim.cgi?id=609442) |  |  |
|  |  |  |  |
| ***OTHER GENES*** |  |  |  |
| *TECTORIN, ALPHA; TECTA* | [*602574](http://www.ncbi.nlm.nih.gov/entrez/dispomim.cgi?id=602574) | [11q22-q24](http://www.ncbi.nlm.nih.gov/Omim/getmap.cgi?l602574) | TECTA |
| *ZINC FINGER PROTEIN OF CEREBELLUM, 2; ZIC2* | [*603073](http://www.ncbi.nlm.nih.gov/entrez/dispomim.cgi?id=603073) | [13q32](http://www.ncbi.nlm.nih.gov/Omim/getmap.cgi?l603073) | ZIC2 |
