# Supplementary material for: Heterozygous Mutations of FREM1 Are Associated with an Increased Risk of Isolated Metopic Craniosynostosis in Humans and Mice
Source: PLoS Genet. 2011 Sep 8;7(9):e1002278. doi: 10.1371/journal.pgen.1002278 (PMC3169541; doi:10.1371/journal.pgen.1002278)
Supplement: Table S2 — Craniofacial measurements. (DOC) [file pgen.1002278.s002.doc]

**Table S2: Craniofacial measurements**

|  | Head Circumference | Inner Canthal | Outer Canthal | Inter Pupillary |
| --- | --- | --- | --- | --- |
| Proband (Patient 7) | 50.5 (<3rd) | 3.5 (+2SD) | 9.0 (75^th^) | 6.25 (>97^th^) |
| Sibling 1 | 52.2(10-25^th^) | 3.7 (+3SD) | 9.2 (75^th^) | 6.45 (>97^th^) |
| Sibling 2 | 51.8 (25-50^th^) | 3.0 (mean) | 8.8 (25^th^-50^th^) | 5.9 (75^th^) |
| Mother | 53 (10-25^th^) | 3.3(+1 SD) | 9.4 (75^th^ -90^th^) | 6.35 (>97^th^) |
