# Supplementary material for: Heterozygous Mutations of FREM1 Are Associated with an Increased Risk of Isolated Metopic Craniosynostosis in Humans and Mice
Source: PLoS Genet. 2011 Sep 8;7(9):e1002278. doi: 10.1371/journal.pgen.1002278 (PMC3169541; doi:10.1371/journal.pgen.1002278)
Supplement: Table S3 — MLPA probes used for CNV screening of FREM1. (DOC) [file pgen.1002278.s003.doc]

**Table S3:** MLPA probes used for CNV screening of *FREM1*

| **MLPA Primer name** | **5' half-probe sequence** | **3' half-probe sequence** |
| --- | --- | --- |
| *CER1_ex2* | GGTCTGTTCATTTTCCTGGAGCCGCGCAGCACT | CCCATACCTCCTGCTCTCACTGTTTGCCTGCCA |
| *FREM1_ex2* | GGGCACATTGTCAGGACCCCT | CACCCTGCCCCTTGGACTATG |
| *FREM1_ex9* | TGTGATTGAACTGGAGGAGGGGCAG | ACCATCCTGATCCAGGGATCCATGC |
| *FREM1_ex16* | TGTGATTGCTCGCGAACCTCAGCATGGGGTG | GTGAGGAGAGCTGGAGTCACAGTGGATCAGT |
| *FREM1_ex23* | gagcagctgactaactctggctccttccagATAGGGAGG | GACTGGGTTCCTCTCTCCCCTGGCATGAAATGCACTCAG |
| *FREM1_ex31* | GCAAGCACAGCACATGGGAGAAGGGCATTTGGCATCTGCTGCC | CCCAGGGTCTTCCTCATCCACCACTTCTGGTTCCTTTCATCTG |
| *TTC39B_intr8* | gagcaggaagaaagcagaggacagatggggccattgtgatg | gcctttgctttcttgtttcagtctgtcctggtcccaagccc |
